# PARENTAL ABSENCE AND GRANDPARENT CAREGIVING IN THE WAKE OF THE CONTEMPORARY AMERICAN OPIOID EPIDEMIC

**DOI:** 10.1093/geroni/igz038.2344

**Published:** 2019-11-08

**Authors:** Jessica Y Ho

**Affiliations:** University of Southern California, Los Angeles, California, United States

## Abstract

The contemporary American drug overdose epidemic has wreaked havoc on individuals, families, and communities across the country. As increasing numbers of middle-aged adults become addicted to or die from drugs, grandparents may be called upon to provide care for their grandchildren. However, few studies examine the broader social impacts of the drug overdose epidemic. This paper fills this gap in the literature by examining whether the opioid epidemic has contributed to increased grandparental care provision and coresidence. I use data from the 2000 Census and the 2011-2015 American Community Surveys, and data on drug overdose mortality (a measure of the epidemic’s severity) and socioeconomic indicators. I find consistent and robust associations between increases in drug overdose mortality and increases in: children’s likelihood of experiencing parental absence from the household, grandparent-grandchild coresidence, and grandparental provision of custodial care. Fixed-effect estimates correspond to an additional 158,138 grandparents coresiding with grandchildren, and a 3% increase in the proportion of coresident grandparents who are primarily responsible for their grandchildren. These associations are stronger among younger (aged 30-64) versus older (aged 65+) grandparents. This paper leverages multiple measures and nationally-representative data to demonstrate that increases in precarious family arrangements, vulnerability, and the burden of caregiving among older adults are occurring in response to the opioid epidemic. These findings suggest that the epidemic is placing considerable and unexpected strains on older adults’ financial and emotional resources at a time when they themselves may be requiring care, retiring from the labor force, and experiencing declines in health.

